# EANM recommendations based on systematic analysis of small animal radionuclide imaging in inflammatory musculoskeletal diseases

**DOI:** 10.1186/s13550-021-00820-8

**Published:** 2021-09-06

**Authors:** Erik H. J. G. Aarntzen, Edel Noriega-Álvarez, Vera Artiko, André H. Dias, Olivier Gheysens, Andor W. J. M. Glaudemans, Chiara Lauri, Giorgio Treglia, Tim van den Wyngaert, Fijs W. B. van Leeuwen, Samantha Y. A. Terry

**Affiliations:** 1 Inflammation and Infection Committee EANM, Vienna, Austria; 2 Department of Medical Imaging, Radboud University Nijmegen Medical Center, Geert Grooteplein Zuid 10, 6525 GA Nijmegen, The Netherlands; 3 Department of Nuclear Medicine, General University Hospital of Ciudad Real, Ciudad Real, Spain; 4 Center for Nuclear Medicine Clinical Center of Serbia, Faculty of Medicine, University of Belgrade, 11000 Belgrade, Serbia; 5 Department of Nuclear Medicine and PET Center, Aarhus University Hospital, Aarhus, Denmark; 6 Department of Nuclear Medicine, Cliniques Universitaires Saint-Luc and Institute of Clinical and Experimental Research (IREC), Université Catholique de Louvain (UCLouvain), Brussels, Belgium; 7 Department of Nuclear Medicine and Molecular Imaging, University Medical Center Groningen, University of Groningen Medical Imaging Center, Hanzeplein 1, 9713 GZ Groningen, The Netherlands; 8 Nuclear Medicine Unit, Department of Medical-Surgical Sciences and of Translational Medicine, “Sapienza” University of Rome, 00161 Rome, Italy; 9 Clinic of Nuclear Medicine, Imaging Institute of Southern Switzerland, Ente Ospedaliero Cantonale, Bellinzona, Switzerland; 10 Department of Nuclear Medicine and Molecular Imaging, Lausanne University Hospital and University of Lausanne, Lausanne, Switzerland; 11 Faculty of Biology and Medicine, Università Della Svizzera Italiana, Lugano, Switzerland; 12 Bone and Joint Committee EANM, Vienna, Austria; 13 Antwerp University Hospital Belgium, Edegem, Belgium; 14 Molecular Imaging Center Antwerp (MICA) - IPPON, Faculty of Medicine and Health Sciences, University of Antwerp, Universiteitsplein 1, 2610 Wilrijk, Belgium; 15 Translational Molecular Imaging and Therapy Committee EANM, Vienna, Austria; 16 Department of Radiology, Interventional Molecular Imaging Laboratory, Leiden University Medical Center, Leiden, The Netherlands; 17 Department of Imaging Chemistry and Biology, School of Biomedical Engineering and Imaging Sciences, King’s College London, 4th Floor Lambeth Wing, St Thomas’ Hospital, London, SE1 7EH UK

**Keywords:** Inflammatory musculoskeletal disease, Rheumatoid disease, Preclinical, Positron emission tomography, Single photon emission computed tomography, Molecular imaging

## Abstract

Inflammatory musculoskeletal diseases represent a group of chronic and disabling conditions that evolve from a complex interplay between genetic and environmental factors that cause perturbations in innate and adaptive immune responses. Understanding the pathogenesis of inflammatory musculoskeletal diseases is, to a large extent, derived from preclinical and basic research experiments. In vivo molecular imaging enables us to study molecular targets and to measure biochemical processes non-invasively and longitudinally, providing information on disease processes and potential therapeutic strategies, e.g. efficacy of novel therapeutic interventions, which is of complementary value next to ex vivo (post mortem) histopathological analysis and molecular assays. Remarkably, the large body of preclinical imaging studies in inflammatory musculoskeletal disease is in contrast with the limited reports on molecular imaging in clinical practice and clinical guidelines. Therefore, in this EANM-endorsed position paper, we performed a systematic review of the preclinical studies in inflammatory musculoskeletal diseases that involve radionuclide imaging, with a detailed description of the animal models used. From these reflections, we provide recommendations on what future studies in this field should encompass to facilitate a greater impact of radionuclide imaging techniques on the translation to clinical settings.

**Supplementary Information:**

The online version contains supplementary material available at (10.1186/s13550-021-00820-8).

## Introduction

Inflammatory musculoskeletal diseases represent a group of chronic and disabling conditions with a major impact on patients’ quality of life and with a high socio-economic impact [1–3]. These conditions evolve from a complex interplay between genetic and environmental factors that cause perturbations in innate and adaptive immune responses. The altered activity in stromal and supportive tissue components ultimately progresses to clinically overt disease [1, 2, 4].

Understanding of the pathogenesis of inflammatory musculoskeletal diseases is not only based on clinical findings but, to a large extent, derived from preclinical and basic research experiments. For example, in translational rheumatoid arthritis research, animal models have proved to be essential in testing hypotheses, novel therapeutics and involved components of the immune system [5]. The previous literature has set out the specifications of available animal models in rheumatoid arthritis [6–8], systemic lupus erythematosus [9–11], scleroderma [12] and osteoarthritis [13]. These reviews provide a rationale for choosing a preclinical model to investigate therapeutic interventions; this could be based on stage of disease development, genetic and immunological background of these models and method of induction of autoimmunity. In addition, the disease phenotype, e.g. mono- versus polyarthritis, first occurrence of symptoms, and initial induction versus re-challenge models, together determines which research questions can appropriately be addressed in a particular disease model. To date, a comprehensive experimental evaluation of available models based on uniform and in-depth analysis, which would provide a framework for researchers to decide which animal model matches their research questions best, is lacking [14–16]. Moreover, no preclinical disease model can fully reflect the complexity of the human disease [17].

In addition to ex vivo histopathological analysis and molecular assays based on blood and joint fluids, as illustrated in detail below, in vivo molecular imaging enables to study molecular targets non-invasively and longitudinally, providing complementary data on pathological processes and potential therapeutic strategies. For inflammatory musculoskeletal disease, tracers have been developed that target specific immunological pathways [e.g. tumour necrosis factor α (TNF α α)], immune cell populations that were hypothesized to play a role in the pathogenesis of inflammatory musculoskeletal diseases (e.g. macrophages) or stromal components involved in tissue destruction or repair (e.g. bone turnover, fibroblasts). Other tracers allow researchers to visualize the presence and dynamics of a therapeutic target [e.g. ^99m^Tc-3PRGD_2_ targeting angiogenesis during treatment with anti-vascular endothelial growth factor (VEGF) monoclonal antibody] or are designed to monitor disease activity (e.g. ^18^F-FDG to measure glucose metabolism). However, similar considerations as in interventional studies arise when interpreting imaging data in small animal models on inflammatory musculoskeletal diseases. For example, in arthritis disease models, K/BxN mice and SKG mice spontaneously develop B- and T-lymphocyte-dependent arthritis, indicating a role for major histocompatibility complex (MHC) class II presentation [18]. However, human TNF α transgenic mouse models also spontaneously develop arthritis, but it was developed in H-2K and H-2B haplotypes, which, in fact, puts the role of MHC class II presentation into question. Furthermore, crossing these transgenic mice with Ras-related C3 botulinum toxin substrate 1 (RAG-1) knockout mice results in erosive arthritis, but, again, without involvement of B- or T-lymphocytes [19]. It is therefore still questionable which preclinical models accurately depict the development of arthritis in humans, those with or without B- or T-lymphocyte involvement? Equally, the RAG-1 knockout model displays high titres of rheumatoid factor and auto-antibodies specific for type II collagen and 70 kilodalton heat shock protein (hsp-70), with no antibodies for double-stranded DNA, resembling lupus. On the contrary, interleukin 1 beta (IL-1β receptor antagonist-deficient mice generated on a BALB/c background) also develops antibodies against type II collagen, but, unlike the TNF α transgenic mouse, not IgM rheumatoid factor; they develop antibodies against double-stranded DNA instead [20]. These examples demonstrate that different disease phenotypes were generated when pleiotropic cytokines such as TNF α and IL-1β were modulated. Such challenges illustrate that developing molecular imaging tracers which specifically target immune cell populations or inflammatory mediators requires understanding of the characteristics of small animal models for interpretation, validation and translation of the imaging findings.

The large body of preclinical studies in inflammatory musculoskeletal diseases is in remarkable contrast with the limited role of molecular imaging in informing and influencing clinical practice and guidelines in these diseases. Our aims are therefore to (1) systematically review the literature on molecular radionuclide imaging studies in preclinical models of inflammatory musculoskeletal diseases and (2) stimulate discussion on the most appropriate use of molecular imaging tools in the field of inflammatory musculoskeletal diseases. In this EANM-endorsed position paper, we thus performed a systematic review of the studies in inflammatory musculoskeletal diseases that involve radionuclide imaging, with a detailed description of the animal models used. From these reflections, we provide recommendations on what future studies in this field should encompass to facilitate a greater impact of radionuclide imaging technique on translating insights in disease and novel treatments to clinical settings.

## Methods

### Search strategy

To identify all relevant publications, a systematic search was performed using the databases PubMed and Cochrane Library for the period 5 March 2009 to 4 February 2020. The following search terms were used: (“animal” OR “preclinical” OR “pre-clinical” OR “experimental” OR “rat” OR “rats” OR “mouse” OR “mice” OR “rabbit” OR “rabbits”) AND (“imaging” OR “PET” OR “SPECT” OR “SPET” OR “tomography”) AND (“arthritis” OR “osteoarthritis” OR “scleroderma” OR “systemic sclerosis” OR “gout” OR “SLE” OR “lupus” OR “rheumat*”).

### Selection process

Three reviewers (ST, EN, EA) independently screened all potentially relevant abstracts for eligibility obtained from the database search. Both studies with and without intervention were included; additional inclusion criteria were:

- Original data, i.e. no reviews, editorials, letters, and comments.
- The study investigated the performance of preclinical PET, PET/CT, SPECT or SPECT/CT systems.
- The study involved mouse, rat or rabbit species.

Full-text articles of these selected records were obtained and reviewed; in this step, records were excluded when no English version of the manuscript was available or when preclinical imaging studies followed radioactive drug or induction agent biodistribution rather than disease processes. Differences in judgment were resolved by consensus.

## Results

### Search results

The literature search generated a total of 1290 records, of which 1203 were excluded based on the initial screening of the abstract. Main reasons for exclusion were (a) no original study (*n* = 144), (b) no SPECT or PET imaging involved (*n* = 787), (c) no relevant preclinical model (*n* = 12) or (d) a combination of previous factors (*n* = 170). The full text of the remaining 87 records was screened for eligibility. An additional 6 abstracts were excluded because of (a) no full text available in English (*n*  = 1) or (b) no preclinical radionuclide imaging of disease, instead imaging of drug, induction agent or another process (*n* =  5) (Additional file 1: Figure S1). A total of 81 studies were included in this review. The Cochrane Library search yielded no relevant additional results. No additional records were identified from references in the selected articles.

Other imaging modalities used in preclinical models (excluding radionuclide PET/SPECT) were microCT and X-ray (n = 405), optical imaging techniques (*n* = 251), MRI (*n* = 144), ultrasound (*n* = 36), photoacoustic imaging (*n* = 13), electron microscopy (*n* = 9), spectral CT (*n* = 6), atomic force microscopy (*n* = 3) and fluorometric imaging (*n* = 1). Several studies had more than one alternative imaging modality.

The animal models used in the 81 studies included in this review were, respectively, a mouse model (*n* = 44), rat model (*n* = 31) and rabbit model (*n* = 6). In Additional file 2: Tables S1, Additional file 3: Tables S2, Additional file 4: Tables S3, a data summary of the selected reports is provided, including type of disease, induction method, strain, age and gender of animals, imaging study size and design, imaging target and modality, radionuclide, tracer, presence or absence of baseline and follow up imaging, correlative outcome measure, whether imaging was of whole body or joints only and main findings.

Notably, no radionuclide imaging studies meeting the inclusion criteria were reported for preclinical models of scleroderma, gout and lupus.

### Strains and triggers of inflammatory musculoskeletal disease

**Fig. 1:**
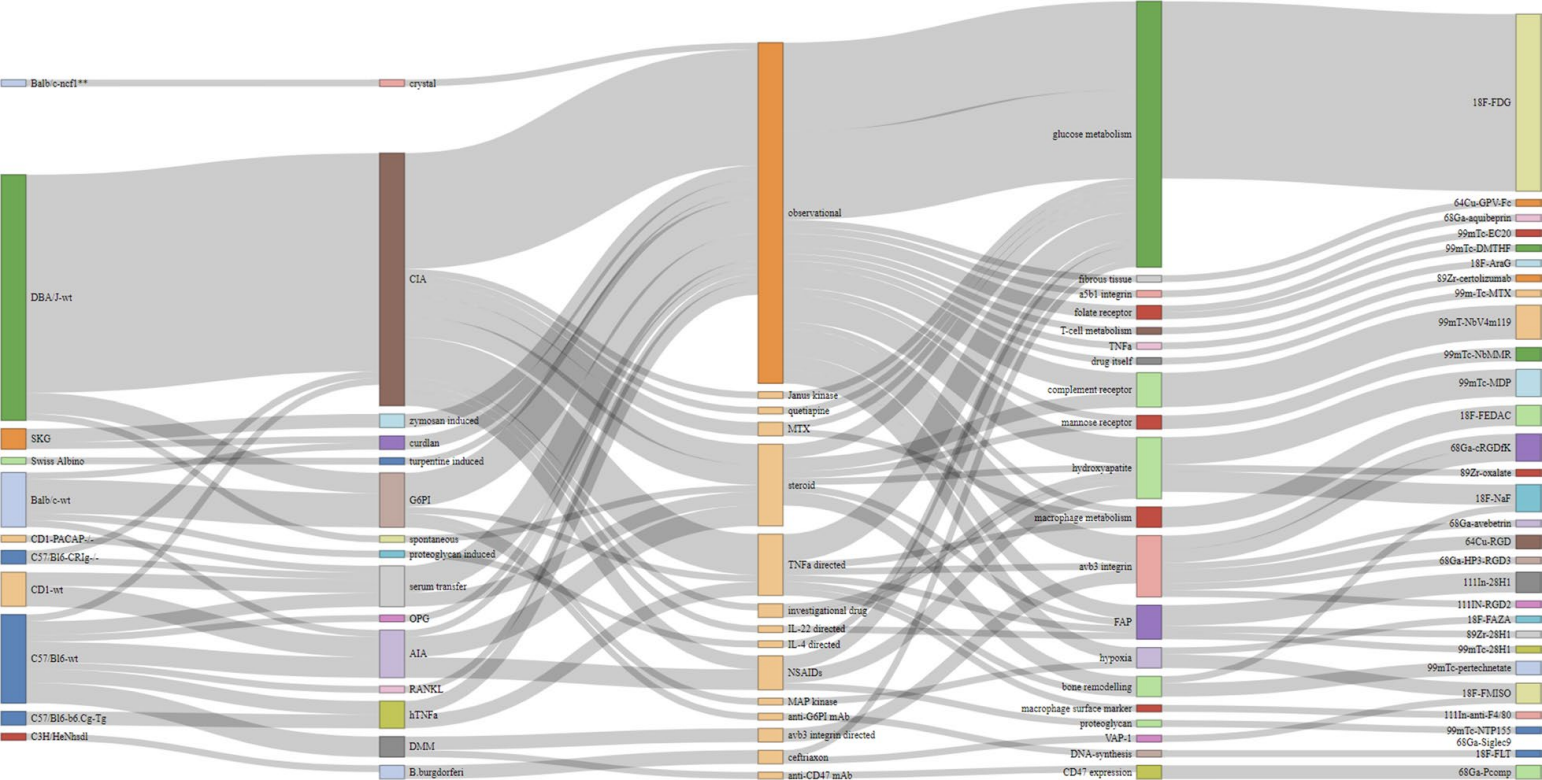
Sankey diagram illustrating the landscape of imaging studies performed in mouse models of inflammatory musculoskeletal diseases by summarizing the interconnections between used mouse models, inflammatory triggers, study designs, imaging targets and tracers. Each line corresponds to one experimental study arm. Apart from dominant models DBA/J-wt and C57/Bl6-wt and collagen-induced arthritis (CIA) as trigger in observational studies, there is a large heterogeneity in terms of triggers, interventions, imaging targets and tracers, resulting in a manifold of single studies with unique combinations. Interactive version of this figure is available at ??? (to be hosted on journal website)

**Fig. 2:**
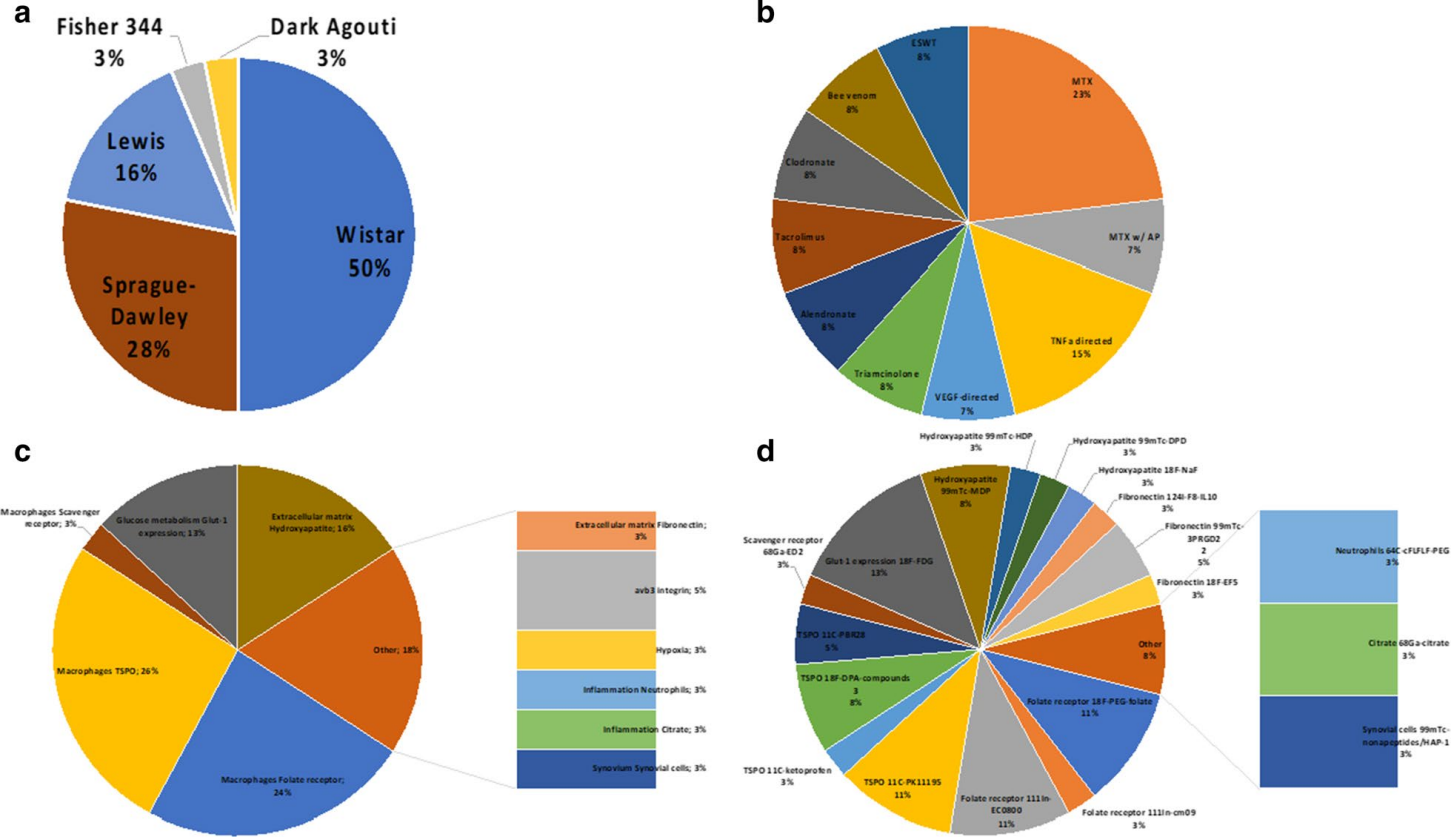
Overview of rat models used (**a**), the type of interventions reported (**b**) and the imaging tracers and targets described in these studies (**c**, **d**)

**Fig. 3:**
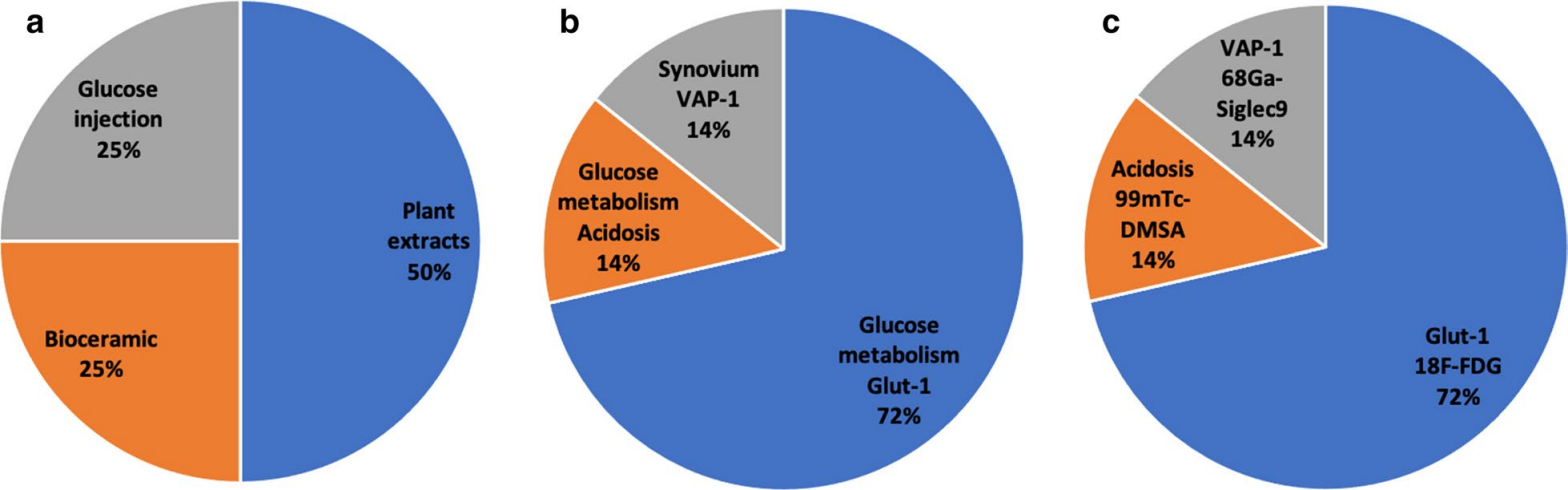
Overview of the type of interventions reported in New Zealand White rabbits (**a**) and the imaging tracers and targets described in these studies (**b**, **c**)

In mouse studies, wildtype DBA/J, C57/Bl6 and BALB/c mice were the most commonly used strains, or variants with specific mutations in relation to the pathological process that is under investigation in these studies (Fig. 1). The variation in rat models is much less and dominated by Wistar, Sprague–Dawley and Lewis strains, but no studies using genetically engineered variants were included (Fig. 2), although transgenic human leukocyte antigen B27 (HLA-B27) rat models are available [21]. All included studies on rabbit models used New Zealand White rabbits (Fig. 3).

Across the three most commonly used strains, collagen-induced arthritis (CIA) and glucose-6-phosphate isomerase (G6PI)-induced arthritis were most prevalent, with the combination of DBA/J mice with CIA ranking first (*n* = 17 studies). In general, the variety in triggers, whether or not being in combination with a specific mouse strain, resulted often in unique experimental set-up in 10 out of 17 analysed studies in mice (Fig. 1), which precludes comparative analyses. Rheumatoid arthritis was the most prevalent disease under investigation, while osteoarthritis was studied in only one report [22]. This interventional study in C57Bl/6 mice in which osteoarthritis was induced through destabilization of the medial meniscus used [^68^ Ga]Ga-c(RGDfK) PET imaging to assess response to treatment with an anti-CD47 antibody or αvβ3 integrin antagonist or focal adhesion kinase (FAK) inhibitor.

In contrast with studies in mice, more osteoarthritis research was carried out in rat models. Out of these 31 studies, 11 studies included osteoarthritis models and 21 studies used a rheumatoid arthritis model, while one study included both arthritis models [23]. As in the mouse models, CIA proved a popular method to induce rheumatoid arthritis in Lewis or Sprague Dawley rats (Fig. 2). Rheumatoid arthritis in rats was also often caused by injection of methylated bovine serum albumin (mBSA) in Wistar rats. For the induction of osteoarthritis, several options without clear superiority were available, including mechanical induction (damaging bone), use of mono-iodoacetate (MIA) and locally injected papain with exercise of rats to put pressure on affected joints and increase arthritic symptoms and scores (Fig. 2). Only the studies describing papain-with-exercise models of arthritis were consistently reported in Wistar rats. The latter can be explained since these four studies were carried out by the same research group [24–27].

The rabbit models consisted mostly of lipopolysaccharide (LPS)-induced arthritis (3 studies from the same group), *Staphylococcus aureus* (1 study), PHA (1 study)-induced arthritis or autologous blood induced arthropathy (1 study).

**Fig. 4:**
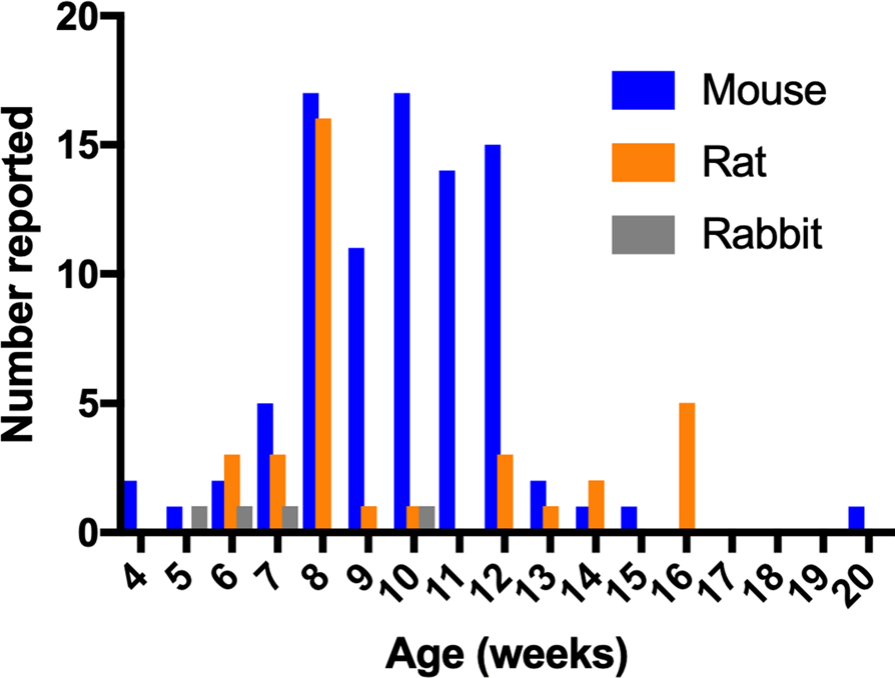
Age distribution of animals used in imaging inflammatory musculoskeletal studies. Seven studies did not report the ages of animals used

Mouse models mostly consisted of young mice mainly between 8 and 12 weeks of age, but with a broad range from 4 weeks up to 20 weeks of age. Notably, 7 studies did not report the age of the mice used (Fig. 4). Rats were mostly used between 8 weeks (or 200 g) to 16 weeks. For rabbit models, age was reported in only two studies, ranging from 5 to 10 weeks, or in body weight (four studies), ranging from 1.5 to 3.8 kg.

**Fig. 5:**
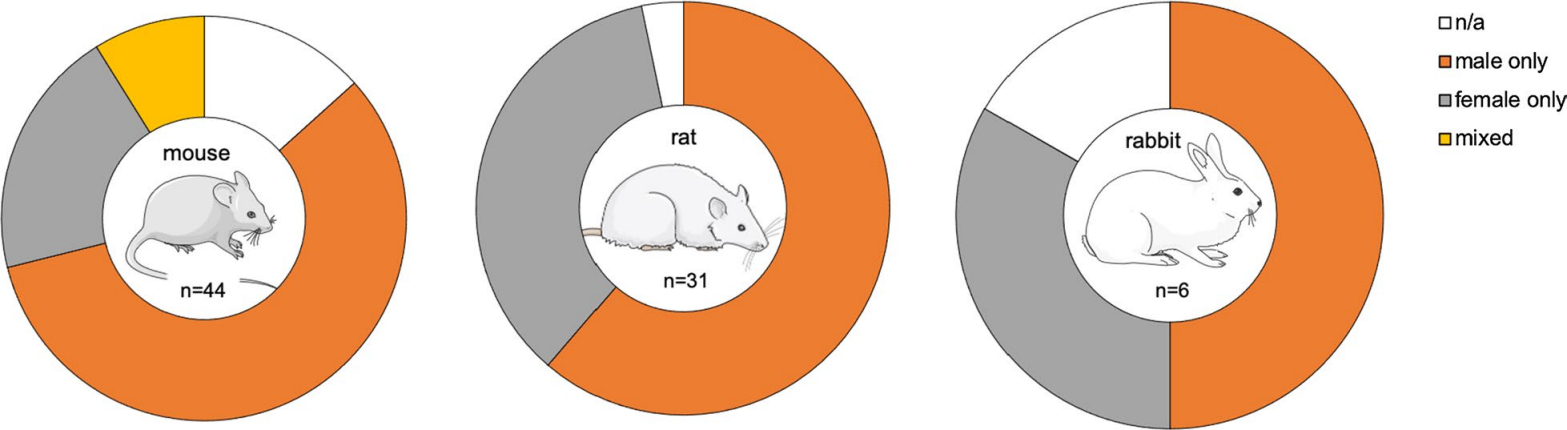
Sex distribution of animals used in imaging inflammatory musculoskeletal disease research

Across different species, male subjects were mostly used, and mixed sexes were only reported in mouse models (Fig. 5). Notably, the information on sex was not available in 6 mouse, 1 rat and 1 rabbit model.

### Study intervention

Of the 44 publications using mouse models, 50% were purely observational (Fig. 1). As part of the remaining 22 studies, a total of 27 interventional studies were carried out with imaging used to assess response to therapies including corticosteroids (e.g. dexamethasone, prednisolone or prednisolone encapsulated in liposomes, n = 6), anti-tumour necrosis factor α TNFα; n = 4), non-steroidal anti-inflammatory drugs (NSAIDs; n = 3) or ceftriaxone (*n* = 2).

61% of the studies in rat models were observational (Fig. 2); in the interventional studies, there was a large variety in therapeutic interventions: 10 different interventions in 13 study arms, with methotrexate (*n* = 3) and TNFα-directed (*n* = 2) treatments mostly used. Two studies on rabbit models were observational, and the remaining 4 investigated the effects of bioceramics, plant extracts or glucose injections (Fig. 3).

### Imaging targets and tracers

Imaging targets and tracers in mouse models were highly variable, with glucose metabolism being targeted in 20 of 55 reported study arms (Fig. 1). Imaging extracellular matrix components (e.g. targeting hydroxy apatite, fibrous tissue or proteoglycans) (*n* = 10) and macrophages (e.g. targeting mannose receptor or cell surface markers) (*n* = 8) were also common targets. Radionuclide imaging tracers also varied although [^18^F]FDG was most prevalent (*n* = 20), followed by sodium[^18^F]fluoride (*n* = 4) and anti-fibroblast activation protein antibody 28H1 (*n* = 3). Five study arms targeted αvβ3 integrin, each using a different RGD peptide or small molecule (avebetrin). Tracers that acted as controls, e.g. radiolabelled isotype control antibodies, were not included. In rat models, an opposite trend was observed (Fig. 2); the dominant target was macrophages (*n* = 20/38), either targeted by tracers specific for the folate receptor (*n* = 9/38 study arms, exploiting 4 different tracers), mitochondrial metabolism (*n* = 10/38, 4 different tracers) or the scavenger receptor CD163 (*n* = 1 study). In rabbit models, most studies used [^18^F]FDG to target inflammation-related glucose metabolism (*n* = 5/7 study arms) (Fig. 3).

### Complementary methods to assess disease outcome

**Fig. 6:**
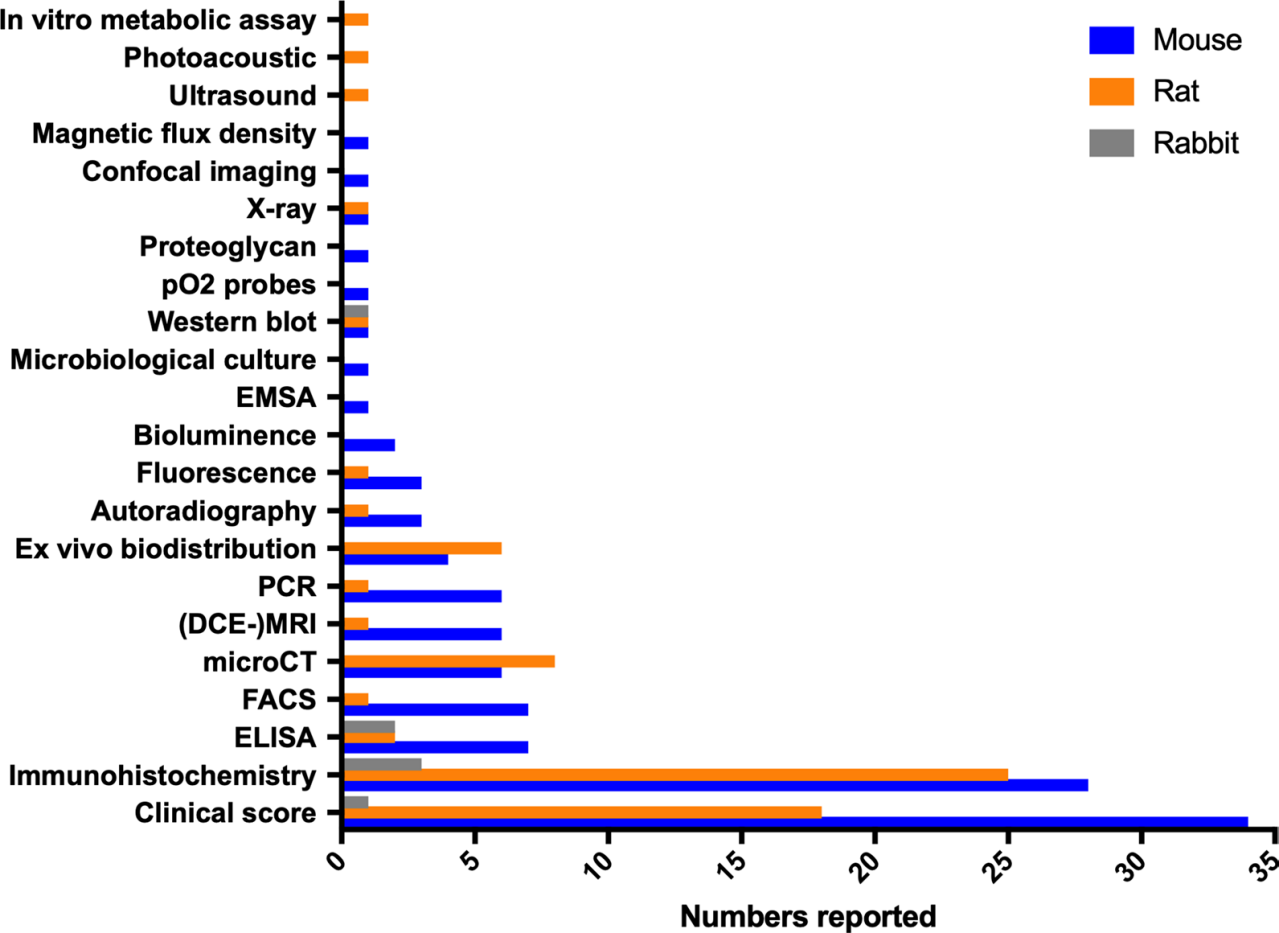
Modalities employed as read-out for imaging findings. EMSA stands for electrophoretic mobility shift assay

**Fig. 7:**
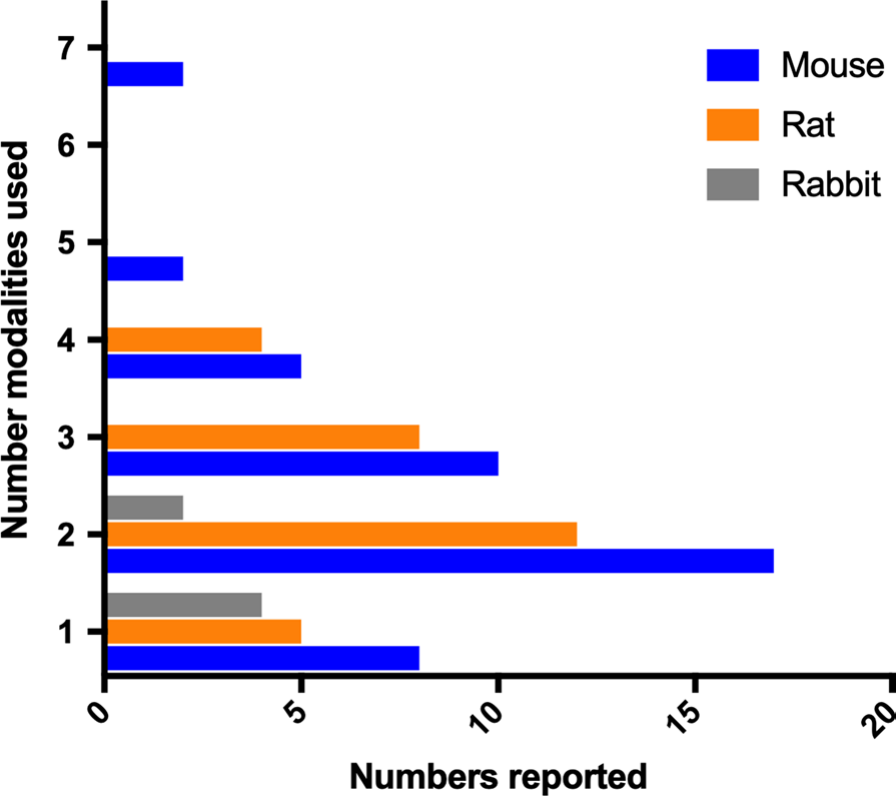
Number of read-outs used per study in preclinical inflammatory musculoskeletal research

In preclinical models, clinical scores, such as paw or joint size by calliper or visual scoring of paw inflammation, taking into account both joint size and colour/soreness, and immunohistochemistry (IHC) were by far the most commonly reported read-outs for complement imaging findings, followed by flow cytometry, enzyme-linked immuno-sorbent assay (ELISA) and polymerase chain reaction (PCR). Few studies assessed arthritis scores through another imaging modality, e.g. microCT, MRI or ultrasound imaging (Fig. 6). Figure 7 shows that in the majority of studies included up to 3 methods complementary to radionuclide imaging was used to assess disease outcome.

### Inclusion of whole body images in publications

Unfortunately, only 64% of the forty-two mouse model studies and only 33% of the thirty rat model studies which included images in the manuscript were whole-body images. This is considered a caveat when it comes to assessing background signal and in vivo distribution of the tracer.

## Discussion

This systematic review describes current molecular imaging tools in place for preclinical research on inflammatory musculoskeletal diseases. Although it is imperative to understand the characteristics of existing models, as reviewed previously [28, 29], it is beyond the scope of this review to provide an in-depth discussion on what model should be employed for which research question. Instead, we reflect on current practice and provide recommendations that should facilitate and divulge the use of molecular imaging tools to further progress the field of inflammatory musculoskeletal diseases [30, 31], as also previously attempted in a recently published consensus [32].

In general, the number of analysed studies using rabbit models was very limited and the observations and recommendations below are therefore based mainly on the analyses of mouse and rat studies. Also, despite the potential of large animal models to bridge preclinical research in rodent models to human studies [33, 34] and to enable advanced quantification methods/tools (e.g. repeated blood sampling possible to aid pharmacokinetics), their current use appears to be limited. This might be due to the complexity in terms of housing, longer life spans of larger animals; it could, however, provide an opportunity: joint size and cartilage thickness are more similar to humans. Bone remodelling in sheep, pigs, dogs closely resembles humans in terms of tissue macro- and microstructure and composition, biochemical properties and mineral density. Moreover, these large animals (and rabbits) need scanning on clinical PET and SPECT systems, whereas small animal PET and SPECT systems have fairly different technical specifications that hamper direct translation of preclinical imaging findings to clinical systems. Equally, the acute nature of inflammation induced in most young animals and the fact that no one mechanism and model can fully represent chronic arthritis found in humans also throw into question the translatability of findings to the clinic.

Another general comment is no studies provided a statistical substantiation of the number of animals used. The ideal number of animals should be limited for ethical reasons, but high enough to ensure an accurate discriminative power. Particularly the latter is in general not stated or discussed, although it would have been considered when filing work protocols at local preclinical ethics committees. A potential source of bias in the selection of studies for analyses is the general omission that studies with negative findings tend to be less often reported [35]. Although a few publications report their negative findings in the field of rheumatoid diseases, e.g. [36], it is most likely that negative imaging studies in also this field are underreported. Lastly, a systematic assessment of the quality of studies, similar to the Quality Assessment on Diagnostic Accuracy studies (QUADAS-2) tool for evaluating clinical studies [37], was not possible as many reports lacked sufficient details to assess all items and compare studies.

We found large heterogeneity with respect to the genetic background, age and sexes of the strains used, often resulting in unique combinations of strains, model of inflammation and tracer used by a research group to target a pathological pathway. Apart from genetic differences as mentioned above, these differences might also concern pathophysiological mechanisms under evaluation, such as fibrosis [38] and angiogenesis [39], and therefore deserve specific attention in tracer development. In our analysis, only a minority of studies used 2 strains to substantiate their findings, 5/44 mouse studies [40–44] and 1/31 rat studies [45].

We recommend validating the diagnostic accuracy of a tracer in different strains and considering include both sexes. Acknowledging the amount of work associated with setting-up models (regulatory issues, licenses, animal welfare office), building a standard of reference and gaining expertise/experience in a particular model, it can be challenging to house multiple models per research group. Hence, we would like to suggest that research groups, as an alternative, collaborate with partners, who are acquainted with a complementary model. Such collaborative multi-centre studies would not only avoid heterogeneity in terms of housing, diet, stress and methods of anaesthesia [46–48], but could also enhance the applicability of the imaging tracers when tested in multiple disease models. As each institute has its unique facilities and each research groups has its expertise, we encourage collaborations via existing platforms (e.g. European infrastructure for translational medicine EATRIS, https://eatris.eu) or via scientific communities such as the EANM to combine these complementary capacities and jointly increase the impact of our research.

Although 14/81 studies compared different imaging tracers [42, 49–61], quantitative details on the sensitivity, diagnostic accuracy and resolving power of a molecular imaging tracer to interrogate a particular pathophysiological feature are insufficient to conclude on the further use of these tracers. Instead, multiple proprietary tracers are developed for the same target. For example, five studies exploited a tracer targeting α_v_β_3_ integrin, two interventional studies [52, 56] and three observational studies [62–64]. Together, these studies were on the background of DBA/1 mice or C57Bl/6 mice [39], and each used a different trigger to induce inflammation. Thus, although five individual studies were reported, the question how to non-invasively target α_v_β_3_ integrin expression best remains unanswered. It appears that, as a scientific community, we rather design and study a new tracer that targets the same process, than put effort in proceeding towards clinical translation or addressing pressing questions in the field of drug development.

Moreover, validation of the diagnostic accuracy of a tracer is in most studies limited to immunohistochemistry and clinical score. Immunohistochemistry is a powerful tool, as it preserves the spatial information of tissue components, but rarely, or never, did groups report the use of (micro-)autoradiography to directly link radioactive signal intensities to specific tissue markers that are subsequently detected by co-registered IHC stainings. Other high-throughput molecular data modalities are hardly employed to cross-validate imaging findings, e.g. CyTOF metabolomics or proteomics. Also, none of the studies used radioactive flow cytometry to validate targeting of specific immune cell populations, which would be highly informative given the mobile and dynamic nature of immune cells. Fluorescence-activated cell sorting (FACS) and ELISA are the workhorse methods in the domains of immunological in vitro and preclinical studies. These immunological assays allow a quantitative in-depth analysis of the current functional status of particular cell populations, receptors or metabolic pathways at great sensitivity. These functional assays of isolated cell populations are complementary to the data on whole-body localization and disease staging derived from in vivo imaging. Cross-validating imaging findings with functional immunological assays would not only aid in the interpretation of imaging but potentially also in a better understanding its limitations. For example, several molecular imaging tracers have been designed to target the presence of macrophages at various stages of disease [65]. However, macrophages are highly plastic and long-lived cells with a plethora of functions in inflammatory responses ranging from inflammatory and antigen-presentation to immune suppressive or wound healing phenotypes [66, 67]. Thus, merely demonstrating macrophages in vivo using imaging does provide information on the presence, localization and dynamics of these cells, but not on the functionality per se. The fact that only 16% and 3 of mouse and rat studies, respectively, used FACS and 16% and 6% of mouse and rat studies, respectively, used ELISA as a validation tool showcases the lack of methodological sharing between imaging scientists and immunologists. We would therefore encourage imaging scientists to consider incorporating immunological assays in their future studies.

Given the large and growing variety in potential models [68], we recommend defining a relevant outcome measure in inflammatory musculoskeletal diseases in consensus, involving multiple stakeholders, e.g. clinicians, pharmaceutical companies and regulatory bodies, on the relevant pathophysiological endpoints.

In relation to the abovementioned comment, only few studies report on the continuation to clinical applications based on their preclinical work. In contrast, many reports conclude remarking that a particular tracer indeed visualizes the disease process, but refrain from commenting on the respective sensitivity, specificity, good manufacturing practices (GMP) compatibility or other tracer features relevant for further translation.

Many parallels can be drawn from imaging applications in the domain of oncological and immune oncological research, as there is also a pressing need to interrogate the dynamics and in vivo distribution of the immune system when therapeutically targeted. Aspects of the previously proposed roadmap in immune oncology could be implemented to push molecular imaging as an indispensable biomarker tool for inflammatory musculoskeletal disease research [69].

In the reported studies, raw acquisition data or analytical data on, for example, volume-of-interest delineation and image quantification were largely lacking. None of the reported studies have reported having stored their data in an open access repository. In addition, most studies report mean ± standard deviation of their experimental data, instead of individual data points even though small numbers of animals per study condition are investigated.

We strongly recommend gearing up, together with other ‘big data’ disciplines such as genetics, biologists and immunologists, and endorse the findability, accessibility, interoperability and reuse (FAIR) principles [70], working towards a widely shared open access policy. This not only calls out to individual research groups, but also accosts our scientific communities, our editors, our funding agencies and regulatory bodies. Such accessible repositories could facilitate the re-use of imaging data for retrospective comparisons or smarter design of trials with re-used data for control arms, which addresses the comments above on the lack of comparative studies. Moreover, as the FAIR principles should apply not only to ‘data’ in the conventional sense, but also to the algorithms for co-registration and quantification, we envision that such efforts also serve faster and easier exchange of expertise regarding immunological assays and technologies that can be used to validate imaging findings (Box 1).

**Box 1:** EANM recommendations on the use of animal models in inflammatory musculoskeletal diseases

Validate the accuracy of your tracer in multiple strains, and consider model-dependent influence of sexes, age and arthritis-inducing trigger
Incorporate complementary immunological assays for cross-validating imaging findings, which will aid in the interpretation as well as better understanding tracer limitations
Define a relevant outcome measure in inflammatory musculoskeletal diseases in consensus, involving multiple stakeholders, e.g. clinicians, pharmaceutical companies, and regulatory bodies, on the relevant pathophysiological endpoints
Endorse the FAIR principle of your molecular imaging data in service of exchange of expertise across disciplines
Use more frequently a large animal model (sheep, pig, dog) since data from such a model are more easily translated to humans
Consider collaborative multi-centre studies, so that expertise in different disease models is combined
Present a statistical substantiation for the number of animals that have been used in your study
Report negative findings
Report whether your preclinical study will be followed up by a further study in humans

## Liability statement

This guideline summarizes the views of the EANM Inflammation and Infection Committee. It reflects recommendations for which the EANM cannot be held responsible. The recommendations should be taken into context of good practice of nuclear medicine and do not substitute for national and international legal or regulatory provisions.

## Supplementary Information

**Additional file 1.Figure S1.** CONSORT flow chart of selecting studies on PET and/or SPECT imaging in mouse, rat or rabbit preclinical models of inflammatory musculoskeletal diseases.

**Additional file 2.Table S1.** Summary of imaging studies using mouse models.

**Additional file 3.Table S2.** Summary of imaging studies using rat models.

**Additional file 4.Table S3.** Summary of imaging studies using rabbit models.

## Data Availability

Available upon request.
