# Supplementary material for: EANM recommendations based on systematic analysis of small animal radionuclide imaging in inflammatory musculoskeletal diseases
Source: EJNMMI Res. 2021 Sep 6;11:85. doi: 10.1186/s13550-021-00820-8 (PMC8421483; doi:10.1186/s13550-021-00820-8)
Supplement: Supplementary file 1 — Additional file 1.Figure S1. CONSORT flow chart of selecting studies on PET and/or SPECT imaging in mouse, rat or rabbit preclinical models of inflammatory musculoskeletal diseases. [file 13550_2021_820_MOESM1_ESM.docx]

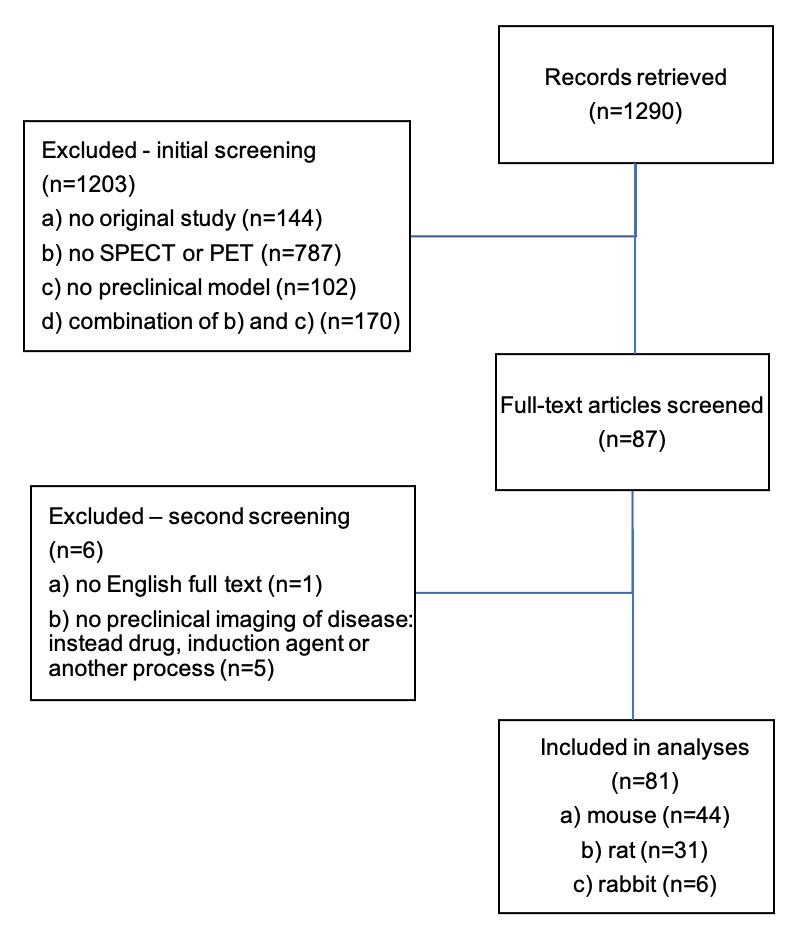


Figure S1: CONSORT flow chart of selecting studies on PET and/or SPECT imaging in mouse, rat or rabbit preclinical models of inflammatory musculoskeletal diseases.
