# Supplementary material for: EANM recommendations based on systematic analysis of small animal radionuclide imaging in inflammatory musculoskeletal diseases
Source: EJNMMI Res. 2021 Sep 6;11:85. doi: 10.1186/s13550-021-00820-8 (PMC8421483; doi:10.1186/s13550-021-00820-8)
Supplement: Supplementary file 2 — Additional file 2.Table S1. Summary of imaging studies using mouse models. [file 13550_2021_820_MOESM2_ESM.docx]

| **Table S1.** Summary of imaging studies using mouse models | | | | | | | | | | |
| --- | --- | --- | --- | --- | --- | --- | --- | --- | --- | --- |
| Author and DOI | Disease induction type | Strain | Imaging study size | Study intervention | Target | Radionuclides and targeting moeities | Baseline imaging | Time after induction (observational) or intervention | Correlative outcome measure | Main imaging findings |
| Chung et al.  doi:10.1016/j.bbrc.2018.10.083 | Collagen-induced arthritis (CIA) | DBA/J | 3 groups, 4-4-2 | interventional; TNFa antagonist, or MTX or saline i.p. | macrophages, glucose metabolism | 18F  FEDAC, FDG | yes | 1 week, 2 weeks | clinical score | both TNF-antagonist and MTX treatment induced reduction in FDG uptake but not FEDAC |
| Pan et al. doi: 10.1007/s00011-018-1176-1 | CIA | DBA/1J | 4 groups, 8-8-8-8 | interventional; quetiapine or celecoxib per gavage, or control | glucose metabolism | 18F  FDG | no | day 20, 32 and 43 post treatment initiation | ex vivo biodistribution, IHC, EMSA, ELISA, FACS, microCT, clinical score | mean SUV of  quetiapine-treated group was significantly lower than of the CIA and celecoxib-treated groups on day 32 and day 43 |
| Czegley et al. doi: 10.1242/dmm.034041 | Monosodium urate crystal-induced enthesitis | oxidative burst-deficient BALB/c / *Ncf1*** | 2 groups | interventional; monosodium urate crystals | hydroxy apatite | 18F  NaF | no | day 2 and 22 post induction | IHC, DCE-MRI, in vivo calcein labeling | SUVs in Ncf1** mice were elevated until  three weeks after injection of MSU crystals |
| Kwon et al. doi: 10.3389/fimmu.2018.01544 | Curdlan-induced RA-ILD | SKG and BALB/c | 3 groups, 6-3-5 | observational | glucose metabolism | 18F  FDG | no | 20 weeks | IHC, MR, ELISA, FACS | hypermetabolic lesions were observed in the peripheral joints, intestine,  and lung not  detected in BALB/c mice or PBS-treated SKG mice |
| Van der Geest et al. doi:  10.1093/rheumatology/kex456 | CIA | DBA-1/J | 6 groups, 6-6-6-6-6-6 | interventional;anti-IL-22 Ab versus isotype control Ab | fibroblast activation protein | 111In  28H1 | no | 12 days after start treatment | IHC, ex vivo biodistribution | neutralizing IL-22 prior to CIA prevents disease development, but not when neutralized after induction, 28H1 imaging precedes clinical score |
| Chung et al. doi: 10.2967/jnumed.117.200667 | CIA | DBA-1 | 4 groups, 10-4-5-2 | observational | macrophages, glucose metabolism | 18F  FDG or FEDAC | no | 23 and 37 days after start treatment | IHC, ex vivo biodistribution | FEDAC signal increases at earlier timepoint (day 23) with no relation to clinical score, FDG increases at day 37 and correlated with clinical score |
| Papachristou et al. doi10.3892/mmr.2017.8166 | experimental inflammation by s.c. turpentine injection | Swiss Albino | 2 groups, 3-3 | observational | N/A | 99mTc  MTX | no | 2 and 24 hrs post-injection | ex vivo biodistribution | radiolabelled MTX accumulates at sites of inflammation, bones, joints, spinal chord |
| Jeong et al. doi: 10.1016/j.jbspin.2017.11.008 | spondylarthritis, zymosan-induced | SKG | 4 groups, 12-2-12-2 | observational | glucose metabolism | 18F  FDG | no | 12 days post induction | IHC, serum cytokine assay | 18F-FDG uptake was significantly higher in the zymosan-treated mice compared with controls |
| Siitonen et al. doi: 10.1186/s13075-017-1460-4 | B.burgdorferi arthritis | C3H/HeNhsd | 3 groups, 8-14-4 | interventional, ceftriaxone | vascular adhesion protein-1 | 68Ga  Siglec-9 | no | 4 days post induction, weekly till 7 weeks | IHC, clinical score | Siglec-9 tracer detects arthritis; despite short-term antibiotic treatment, the arthritis persisted |
| Jeong et al. doi: 10.1186/s13075-017-1407-9 | spondylarthritis, zymosan-induced | SKG | 3 groups, 12-12-2 | observational | glucose metabolism | 18F  FDG | no | 8 weeks post induction | IHC, clinical score, cytokines in joint fluid | 18F-FDG uptake was significantly lower in E2-treated mice than  in sham-operated (sham) and ovariectomized mice |
| Pietikäinen et al. doi: 10.1080/03009742.2017.1287306 | systemic B.burgdorferi infection, including arthritis | C3H/HeNhsd | 5 groups, 4-10-4-4-4 | interventional, ceftriaxone | glucose metabolism | 18F  FDG | no | 4 days post induction, weekly till 7 weeks | IHC, microbiological culture, clinical score | FDG identifies sites of B.burgdorferi infection and responds to antibiotic treatment |
| Franc et al. doi: 10.1177/1536012117712638 | AIA | Balb/c | 2 groups, 3-3 | observational | T-cells | 18F  9-b-D- arabinofuranosylguanine (AraG) | no | day 6 and 20 post induction | IHC, FACS | AraG signal increases with activation status of T-cells |
| Hoffmann et al. doi: 10.1038/s41598-017-02389-6 | glucose-6-phosphate isomerase (G6PI)–induced arthritis | DBA-1 | 2 groups, 10-10 | observational | hydroxy apatite | 18F  fluoride | yes | day 10-14-18-24-35 post induction | clinical score | Fluoride and microCT identify early changes in bone structure in arthritis |
| Fuchs et al. doi: 10.2967/jnumed.116.185934 | G6PI-induced arthritis | Balb/c | N/A | observational | hypoxia | 18F  FMISO and FAZA | no | day 1-3-6 post-induction | clinical score, IHC, FACS, PCR, western blot, autoradiography, pO2 probes | FAZA and FMISO assess hypoxia in inflammation models |
| Mitra et al. doi: 10.1111/cei.12926 | CIA | DBA/1J | 2 groups, 10-5 | interventional, anti-TNF-α antibody | glucose metabolism | 18F  FDG | yes | day 0-28-45(pre-treatment)-56(post-treatment) | clinical score, IHC | FDG PET is a tool to monitor the therapeutic effects in vivo |
| Imberti et al. doi: 10.1021/acs.bioconjchem.6b00621. | K/BxN serum transfer | C57Bl/6 | 4 groups, 3-3-3-3 | observational | avb3 integrin | 68Ga  HP3-RGD3 | no | day 8 | clinical score | HP3-RGD3 enables assessment of αvβ3 integrin receptor expression in vivo |
| Zheng et al. doi: 10.1038/srep35966. | CIA and K/BxN serum transfer | C57Bl/6 (WT and CRIg−/−) and DBA/1 | 13 groups, 2-2-2-2-2-2-4-4-4-4-12-10-10 | interventional, dexamethasone | complement receptor of Ig superfamily, mannose receptor, beta-lactamase *Bacillus cereus* (control) | 99mTc  NbV4m119, NbMMR, NbBCII10 (control) | yes | day 34-38-42 for CIA model  day 2-8-15 for STIA model  day 12 for dexamethasone model | clinical score, microCT | NbV4m119 allows specific assessment of inflammation in different arthritis models and upon treatment |
| van der Geest et al. doi: 10.2967/jnumed.116.177931 | CIA | DBA/1J | 2 groups, n=5-5 | Interventional long-circulating liposomes (LCL) containing  prednisolone phosphate | fibroblast activation protein | 99mTc  28H1 | no | day 2-5-9 after treatment | clinical score | 28H1, targeting fibroblasts, can noninvasively monitor the course of CIA |
| Terry et al. doi: 10.2967/jnumed.115.162628. | CIA | DBA/1J | 6 groups, n=10-10-10-10-10-10 | interventional;  anti-TNF-a | avb3 integrin, macrophage, fibroblast activation protein | 111In  RGD2,  anti-F4/80-A3-1,  28H1 | no | N/A | clinical score | 28H1, F4/80-A3-1 and RGD2 can be used to monitor the response to therapy in experimental arthritis |
| van der Geest et al. doi: 10.1016/j.jconrel.2015.04.019 | AIA | C57Bl6/J | 4 groups, n=6-6-6-6 | interventional;  prednisolone (PLP)-containing  PEG-liposomes | glucose metabolism | 18F  FDG | yes | day 3-7-12 | clinical score, IHC | PLP-containing liposomes reduce FDG accumulation |
| Lin et al. doi: 10.1159/000374026. | proteoglycan-induced arthritis (PGIA) | Balb/c | 2 groups, n=40-40 | interventional;  intra-articular IL-4 | glucose metabolism | 18F  FDG | no | week 10 and 14 | clinical score,  FACS,  RT-qPCR | uptake of tracer was significantly reduced in IL-4-  treated mice at 14 weeks |
| Laverman et al. doi: 10.2967/jnumed.114.152959. | CIA | DBA/1J | 2 groups, n=3-3-3-3 | observational | fibroblast activation protein, glucose metabolism | 111In,  89Zr  18F  28H1, FDG | no | day 24-26 | clinical score, IHC | 28H1 visualized arthritic joints, SPECT preferred over PET |
| Khairnar et al. doi: 10.2967/jnumed.114.151415. | AIA | CD1 | 4 groups, n=5–8 per group | interventional; meloxicam | proteoglycan, hydroxy apatite, glucose metabolism | 99mTc,  18F  NTP15-5,  MDP,  FDG | no | NTP15-5: days 3, 14, and 28  MDP: days 4, 15 and 29  FDG: days 4, 15 and 29 | clinical score, IHC, proteoglycan content | NTP 15-5 showed  specific tracer accumulation within RA joints |
| Irmler et al. doi: 10.1186/ar4670 | G6PI-induced arthritis | DBA/1 | 1 group, n =3-6 per time point | observational | hydroxy apatite | 18 F  NaF | yes | day 14, 28 and 50 | 6clinical score, microCT, IHC | 18 F-fluoride signaling at different stages of G6PI-induced arthritis was significantly correlated with the degree  of bone destruction |
| Botz et al. doi: 10.1002/art.38772. | K/BxN serum transfer | CD1, PACAP−/− and wild-type (PACAP+/+) | 4 groups, n= 3-3-3-3 | observational | glucose metabolism | 18F  FDG | no | day 4 | clinical score, microCT, fluorescence, bioluminescence, MRI | FDG uptake increased in PACAP+/+ arthritic mice compared to control PACAP+/+ mice but decreased in PACAP-/- environment. |
| Kundu-Raychaudhuri et al. doi: 10.1111/1756-185X.12410. | CIA | DBA/1Jf | 3 groups (per time point), n=18-4-5 | observational | glucose metabolism | 18F  FDG | yes | day 28, 56 | clinical score, IHC | FDG uptake correlates  with different stages of the disease |
| Zheng et al. doi: 10.2967/jnumed.113.130617. | CIA | C57BL/6, DBA/1, CRIg-/- | 3 groups (different models) | observational | complement receptor of Ig superfamily | 99mTc  NbV4m119 | no | day 23 | clinical score,  PCR, IHC | NbV4m119 visualizes joint inflammation in CIA |
| Put et al. doi: 10.2967/jnumed.112.111781. | CIA | DBA/1 | *4 groups (*18-14-8-18) | observational | mannose receptor, beta-lactamase *Bacillus cereus* (control) | 99mTc  NbMMR, BCII10 (control) | no | day 35 | clinical score, FACS | MMR targets macrophages in synovial fluid of inflamed paws, |
| Bender et al.  PMCID: PMC3560477 | CIA | DBA/1 | 4 groups, n=3 -3-3-3 | interventional; ER-886046,  prednisolone or celecoxib | hydroxy apatite | 99mTc  MDP | yes | day 6, 12 and 20 | clinical score, bioluminescence, IHC, X-ray | The compound ER-886046 decreased MDP uptake in paws |
| Fuchs et al. doi: 10.2967/jnumed.112.106740. | G6PI-induced arthritis | Balb/c | 4 groups, n=2-2-2-2 | interventional; glucose-6-phosphate-isomerase–specific antibodies | DNA synthesis | 18F  3’-deoxy- 3’-18F-fluorothymidine (FLT) | no | day 1, 3, 6, and 8 | clinical score, IHC, MRI | FLT targets cell proliferation in arthritic joint inflammation |
| Cha et al. doi: 10.3348/kjr.2012.13.4.450. | CIA | DBA/1J | 2 groups, n=10-3 | observational | glucose metabolism | 18F  FDG | no | day 9 | Fluorescence,  Confocal Laser Scanning Microscopy | chitosan nanoparticles for fluorescence imaging correlated moderately with FDG uptake |
| Vaitilingam et al. doi: 10.2967/jnumed.111.099390. | CIA | DBA/1J | N/A | observational | folate receptor (alpha) - tumour | 99mTc  EC20  DMTHF | no | based on arthritis score | clinical score,  FACS | DMTHF enables selective imaging  tumor in the presence of inflammation |
| Braem et al. doi: 10.1186/ar3772. | spontaneous arthritis | DBA/1 | 2 groups, n=10-12 | interventional; Dexamethasone  or phosphate buffered saline | glucose metabolism | 18F  FDG | no | week 15 and 20 | clinical score,  real-time PCR | FDG uptake decreased in Dexamethasone treated paws. |
| Zheleznyak et al. doi: 10.1007/s11307-011-0512-4. | osteoprotegerin (OPG) or RANKL induced arthritis | C57BL/6 | 2 groups, n=4-4 | observational | alpha-v-beta-3 integrin | 64Cu  RGD | no | day 15 or 11, respectively | IHC, serum analyses | RGD localizes to areas in bone with  increased osteoclast numbers |
| Irmler et al. doi: 10.1186/ar3176. | G6PI-induced arthritis | DBA/1 | 3 groups, n=5-6-42 | interventional; anti-TNF | glucose metabolism | 18F  FDG | no | 0-10 minutes, day 2-6-9-13-21-35 after induction, in different groups | clinical score, IHC, micro-CT | FDG is a feasible method for quantitative assessment of inflammation in G6PI-arthritis |
| Butoescu et al. doi: 10.1186/ar2701. | antigen-induced arthritis | C57Bl/6 | 6 groups, n=5-5-5-5-5-5 | interventional; nanoparticles with superparamagnetic iron oxide and dexamethasone | bone remodelling | 99mTc  pertechnetate | no | at days 1 and 4 after arthritis induction | Clinical score, IHC, magnetic flux density,  fluorescent | presence of an  implanted magnet did not induce a higher 99mTc accumulation  compared with the magnet-free animals |
| Notni et al.  .org/10.1186/s13550-019-0541-6 | CIA | DBA/1JRj | 6 groups, n=3-6-6-6-6-6 | observational | avb3 integrin  a5b1 integrin | 68Ga  avebetrin  aquibeprin | no | at week 1, 2, 3, 4 and 6 after induction | clinical score, IHC, MRI | in advanced RA, a5b1 integrin signal exceeds avb3 integrin signal, and a5b1 integrin signal preceded clinical symptoms of RA |
| Hayer et al. DOI: 10.1002/jbmr.3748 | hTNFtg | C57Bl/6, wt littermate controls | 2 groups, n=6-6 | interventional, anti-TNF antibody | glucose metabolism  bone remodelling | 18F  FDG  fluoride | yes | day 28 after treatment | clinical score, micro-CT, IHC, ELISA rt-qPCR | FDG, but not fluoride, imaging correlated with severity and resolution of bone damage |
| Beziere et al. doi: 10.7150/thno.28892 | G6PI-induced arthritis | Balb/c |  | observational | fibrous tissue | 64Cu  platelet glycoprotein  VI-based ECM-targeting fusion protein (GPVI-Fc) | yes | day 3 and 6 after induction | clinical score, IHC, MRI, auto-radiography | increased uptake in inflamed tissue,  particularly in the RA model |
| Guenthoer et al. doi.org/10.1007/s10787-019-00593-6 | G6PI-induced arthritis | Balb/c | 3 groups, n=12-11-19 | interventional, MAP kinase inhibitors | hypoxia | 18F  FMISO | no | day 3 and 6 after induction | clinical score, IHC | both selective MAP kinase inhibitors were therapeutic, but effect could not be imaged with FMISO |
| Raychaudhuri et al, DOI: 10.1111/1756-185X.13732 | CIA | DBA/1 | 3 groups, n=13-8-3 | interventional, JAK inhibitor | glucose metabolism | 18F  FDG | yes | 1 hour, 5 hour after treatment, and 6 days after treatment | clinical score | FDG PET detects changes in inflammatory activity during JAK inhibition |
| Wang et al. doi.org/10.1172/jci.insight.128616. | destabilization of the  medial meniscus (DMM) | C57BL/6J background, B6.129S2-i*tgb3^tm1Hyn^*/JSemJ, B6.129S7-*Cd47^tm1Fpl^*, 129-*Fyn^tm1Sor^*/J or wt | 6 groups, n=6-6-8-7-6-5 | interventional, anti-CD47 antibody or avb3 integrin antagonist or FAK inhibitor | avb3 integrin expression  CD47 expression | 68Ga  c[RGDfK] or Pcomp | no | 12-20 weeks after induction | clinical score, qPCR, ELISA, IHC | MicroPET/CT imaging of a mouse model showed elevated ligand-binding capacities of  integrin aVb3 and CD47 in osteoarthritic joints |
| Beckford-Vera et al. DOI: 10.1007/s11307-019-01363-0 | transgenic human TNFa induced RA) | B6.Cg-Tg (and C57BL/6 (control) | 3 groups, n=3-3-3 | observational | TNFa | 89Zr  certolizumab | no | at 4, 24,72 and 96 hours post-injection | clinical score | certolizumab accumulated in inflamed joints of transgenic mice that  express hTNFα. |
| Park et al. DOI: 10.1021/acs.molpharmaceut.6b00411 | CIA | DBA/1 | N/A | observational | Glucose metabolism and N/A | 18F  89Zr  FDG  oxalate | no | At day 9 and 10 | Clinical score, IHC, autoradiography | Oxalate monitors inflammatory processes |

Table S1. Summary of imaging studies using mouse models
