# Supplementary material for: EANM recommendations based on systematic analysis of small animal radionuclide imaging in inflammatory musculoskeletal diseases
Source: EJNMMI Res. 2021 Sep 6;11:85. doi: 10.1186/s13550-021-00820-8 (PMC8421483; doi:10.1186/s13550-021-00820-8)
Supplement: Supplementary file 3 — Additional file 3.Table S2. Summary of imaging studies using rat models. [file 13550_2021_820_MOESM3_ESM.docx]

| **Table S2.** Summary of imaging studies using rat models | | | | | | | | | | | |
| --- | --- | --- | --- | --- | --- | --- | --- | --- | --- | --- | --- |
| Author and DOI | Disease induction type | Strain | Imaging study size | Study intervention | Target | Modality | Radionuclides and targeting moeities | Baseline imaging | Time after induction (observational) or intervention | Correlative outcome measure | Main imaging findings |
| Bruijnen et al. Mol pharm doi: 10.1021/acs.molpharmaceut.8b00982 | methylated BSA induced mono-arthritis | Wistar | 4 groups, 4-6-6-6 | observational | fibronectin | PET | I-124  F8-IL10  control KSF-IL10 | no | 10 min and 24 hrs | IHC, ex vivo biodistribution | 124I-F8-IL10 specifically targets extra domain A of fibronectin in joints |
| Chandrupatla et al. doi: 10.1016/j.trsl.2018.04.001 | methylated BSA induced mono-arthritis | Wistar | 8 groups, 3-6 rats | interventional; MTX w/ or w/o alkaline phosphatase | folate receptor | PET | 18F  PEG-folate | yes | day 22 (before AP treatment) and day 40 | IHC, ex vivo biodistribution, fluorescence | [18F]fluoro-PEG-folate PET38,41 and ex vivo tissue distribution  studies proved to be of added value to monitor  the effects of AP and MTX treatment in arthritic rats |
| Chandrupatla et al. doi: 10.1155/2018/8092781 | methylated BSA induced mono-arthritis | Wistar | 3 groups, 4-4-3 | interventional; MTX i.a. vs saline i.a. | folate receptor | PET | 18F  PEG-folate | no | day 6 after last i.a. injection | IHC, ex vivo biodistribution | [18F]fluoro-  PEG-folate could visualize decreased accumulation of the  tracer in the joints. spleen and liver of arthritic rats treated with MTX. |
| De Visser et al. Doi: 10.1177/1947603517738073 | Mechanically-induced osteoarthritis | Wistar | 4 groups, 5-5-2-6 | observational | folate receptor | SPECT | 111In  folate conjugates cm09 or EC0800 | no | 12 weeks post induction | IHC | cm09 (albumin-binding entity) signal increased in osteoarthritis on high-fat diet |
| Chandrupatla et al. doi: 10.1186/s13075-017-1325-x | methylated BSA induced mono-arthritis | Wistar | 3 groups, 4-4-4 | interventional, MTX i.p. | folate receptor | PET | 18F  PEG-folate | yes | day 22 (before MTX treatment) and day 40 | IHC, ex vivo biodistribution | PEG-folate signal identifies arthritic joints and reduces upon MTX treatment |
| Nozaki et al. doi: 10.1007/s11307-016-1039-5 | CIA | Lewis | 8 groups, 4-4-4-4-4-4-4-4 | interventional, anti-TNFa | N/A or macrophages mitochondrial metabolism | PET | 11C  ketoprofen or PK11195 | yes | day 28 | clinical score, IHC, microCT | PK11195 and ketoprofen monitor inflammation and changes upon treatment |
| Wu et al. doi: 10.18632/oncotarget.13953. | CIA | Sprague Dawley | 3 groups, n=5-13-40 | interventional, anti-VEGF mAb | avb3 integrin, hydroxy apatite | Scintigraphy | 99mTc  arginylglycylaspartic acid (3PRGD2), methyl diphosphonate (MDP) | yes | day 15-30 | clinical score, IHC, X-ray | 3PRGD2 imaging detects early rheumatoid arthritis associated synovial angiogenesis |
| Yang et al. doi: 10.1002/jor.23148. | MIA (monoiodoacetate) induced osteoarthritis | Sprague–Dawley | 1 group, n=3 | observational | neutrophil | PET | 64Cu  cFLFLF‐PEG | no | day 5 | IHC | cFLFLF‐PEG‐64Cu specifically binds to Fpr1 and can differentiate arthritic from healthy knees |
| Siebelt et al. doi: 10.1186/s13075-015-0865-1. | papain and running induced osteoarthritis | Wistar | 2 groups, n=20-20 | interventional, triamcinolone (TA) | folate receptor | SPECT | 111In  EC0800 | no | week 6 and 12 | microCT, IHC | TA injections induce macrophage with anti-inflammatory characteristics as monitored through 111n-EC0800 SPECT |
| Huang et al. doi: 10.1097/MNM.0000000000000375. | CIA | Sprague–Dawley | n=8 | observational | avb3 integrin | Scintigraphy | 99mTc  3PRGD2 | no | N/A | clinical score, IHC | 3PRGD2 detects arthritis. |
| Chandrupatla el al. doi: 10.1155/2015/509295. | methylated BSA induced mono-arthritis | Wistar | 7 groups, n=11-3-4-3-6-4-4 | observational | glucose metabolism,  macrophage mitrochondrial metabolism | PET | 18F, 11C  FDG, PK11195 | no | day 6, 19 or 28 | clinical score, IHC, anti-mBSA levels, FACS | Increased [18F]FDG and (R)-[11C]PK11195  accumulation was demonstrated in arthritic knees as compared to contralateral knee |
| Eichendorff et al. doi: 10.1007/s11307-014-0768-6. | CIA | Lewis | 2 groups, n=3-5 | observational | scavenger receptor (CD163) | PET | 68Ga  ED2 | no | days 22 and23, | clinical score, western Blot | ED2 specifically binds CD163 in vivo. The arthritic paws exhibited a low but significant  [68Ga]ED2 uptake |
| Siebelt et al. doi: 10.1016/j.bone.2014.06.009. | papain and running induced osteoarthritis | Wistar | 2 groups, n=20-20 | interventional; alendronate | folate receptor | SPECT | 111In  EC0800 | no | week 6 and 12 | microCT, IHC | ALN treatment reduced tracer uptake in arthritic joints |
| Gent et al. doi: 10.1186/ar4509. | methylated BSA-induced arthritis | Wistar | 3 groups (per tracer), n=5-7-5 | observational | macrophage mitochondrial metabolism | PET | 11C  18F  DPA-713,  DPA-714, PK11195 | no | day 27 | clinical score, IHC | DPA-713 and DPA-714 have improved target-to-background contrast compared to PK11195 |
| Pottier et al. doi: 10.1186/ar4508. | CFA induced arthritis | Dark Agouti | 2 groups, n=6-11 | observational | macrophage mitochondrial metabolism | PET | 18F  DPA-714 | yes | day 20 | clinical score,  IHC | DPA-714 visualizes inflammation in an experimental rodent model of RA |
| Siebelt et al. doi:  10.1016/j.joca.2014.02.003. | papain and running induced osteoarthritis | Wistar | 2 groups, n=20-20 | interventional; tacrolimus | folate receptor | SPECT | 111In  EC0800 | no | week 6 and 12 | clinical score,  microCT, IHC | tacrolimus reduced macrophage activation and EC0800 uptake in arthritic joints |
| Siebelt et al. doi: 10.1186/ar4461. | papain w/ or w/o running induced osteoarthritis | Wistar | 2 groups n=20-20 | observational | folate receptor | SPECT | 111In  EC0800 | no | week 6 and 12 | clinical score, microCT, IHC | Moderate exercise enhances OA progression in papain-injected joints which leads to enhanced EC0800 uptake in arthritic joints |
| Zhang et al. DOI: 10.3760/cma.j.issn.0366-6999.20130726 | CIA | Sprague Dawley | 2 groups, n=8-8 | observational | glucose metabolism | PET | 18F  FDG | no | every 3 days until week 5 | clinical score IHC, MRI | FDG detects RA changes earlier than MR |
| Shao et al. doi: 10.1016/j.nucmedbio.2013.06.008. | model A: local carrageenan injection induced arthritis  model B: heat-inactivated *Mycobacterium butyricum* | Model A: Fisher 344  Model B: Lewis | 4 groups, n=6-4-8-4 | observational | macrophage mitochondrial metabolism | PET | 11C  PBR28 | no | Model A: 3 hours  Model B: 3–4 weeks | clinical score,  IHC | PBR28 is a tracer targeting inflammatory processes involving macrophage activation |
| Rajian et al. doi: 10.1364/BOE.4.000900. | lyophilized *Mycobacterium butyricum* induced arthritis | Lewis | 3 groups, N/A | interventional; anti-TNFa | macrophage mitochondrial metabolism | PET | 11C  PBR28 | yes | day 0 (prior to induction), 20 (prior to treatment) and 35 | clinical score, IHC, photoacoustic imaging, ultrasound imaging | PET imaging follows arthritis progression and therapy response |
| Piscaer et al. doi: 10.1016/j.joca.2013.03.004. | mono-iodoacetate (MIA) induced osteoarthritis | Wistar | C | observational | hydroxy apatite | SPECT | 99mTc  MDP | no | day 2, 14 and 42 | clinical score, microCT  IHC | MDP uptake increases at early timepoints during osteoarthritis |
| Gent et al. doi: 10.1186/ar4191. | methylated bovine serum albumin induced arthritis | Wistar | 3 groups, n=4-8-5 | observational | folate receptor, macrophage mitochondrial metabolism | PET | 18F, 11C  PEG-folate, PK11195 | no | day 27 | clinical score, IHC | folate tracer shows higher contrast due to a lower background signal than macrophage tracer PK11195 |
| Ali et al. doi: 10.1016/j.nucmedbio.2011.01.007. | *Mycobacterium tuberculosis* induced arthritis | Wistar | 2 groups, n=10-10 | observational | synovial cells | Scintigraphy | 99mTc  nonapeptide coupled to HAP-1 | no | based on clinical score | biodistribution | greater  peptide localization of peptides to  inflamed joints following specific conjugation to homing peptides |
| Piscaer et al. doi: 10.1002/art.30363. | Model A-B: osteoarthritis induced by anterior cruciate ligament transection or monoiodoacetate (MIA) injection Model C: antigen induced rheumatoid arthritis (AIA) | Wistar | Model A-B: 3 groups, n=4-4-7  Model C: 1 group, n=2 | interventional; macrophage depletion by clodronate | folate receptor beta | SPECT | 111In  folate | no | Model A-B: week 2-4-9 and 12, week 1-2-4-8  Model C: day 10 | microCT | macrophage activation in experimental OA and RA  can be demonstrated by the folate radiotracer |
| Paquet et al. doi: 10.3233/BME-2010-0632. | *Mycobacterium* wall induced arthritis | Wistar Han | 4 groups, n=2-2-2-2 (per time point) | observational | glucose metabolism | PET | 18F  folate | no | day 1, 2, 3 and 7 after induction | IHC | FDG accumulation reflects inflammatory activity |
| Umemoto et al. doi: 10.1007/s12149-010-0411-9. | anterior cruciate ligament transection  induced osteoarthritis | Sprague-Dawley | n=2 (underwent PET) | observational | hydroxyapatite | PET | 18F  Fluoride | no | week 2, 4, and 8 after induction | clinical score, IHC, ELISA | uptake of fluoride was significantly higher in ACLT knees than sham-operated knees starting at medial locations, in correlation with histological scores |
| Yang et al. doi: 10.1007/s12149-010-0380-z. | carrageenan induced arthritis | Sprague–Dawley | 4 groups, n=3-3-3-3 | interventional; bee venom | hydroxyapatite | Scintigraphy | Tc-99m  DPD | yes | at 4 and 24 hours after  therapy | N/A | bone scintigraphy performed on 4 hours assesses response to bee venom |
| Matsui et al. doi: 10.2967/jnumed.108.060103 | CIA | Lewis | 4 groups, n=3-3-3-3 | observational | glucose metabolism | PET | 18F  FDG | yes | at day 10, 14, and 17 after induction | clinical score, macro-autoradiography, IHC,  3H-FDG in vitro | FDG accumulation in RA reflects proliferating pannus and  inflammatory activity |
| Sperry et al. doi: 10.1002/jor.24581. | mechanical jaw loading | Holtzman Sprague Dawley | 2 groups, n=7-4 (underwent PET) | observational | hypoxia | PET | 18F  EF5 | no | at day 7 after induction | clinical score, IHC, EF5 IHC | EF5 uptake increased after heavy loading of temporo-mandibular joints |
| Kim et al. doi.org/10.1016/j.joca.2019.07.008 | mono-iodoacetate (MIA) induced osteoarthritis | Sprague Dawley | 3 groups, n=3-6-6 | interventional, extracorporeal shockwave therapy (ESWT) | bone remodelling | SPECT | 99mTc  hydroxymethylene diphosphonate (HDP) | no | at 4 weeks after induction | clinical score, micro-CT, IHC, rt-PCR | Lower uptake of HDP in ESWT group, corresponding to improved bone structure on micro-CT |
| Wang et al. doi.org/10.1155/2019/2353658 | CIA | Sprague Dawley | 2 groups, n=22-22 | observational | inflammation  glucose metabolism | PET | 68Ga  18F  citrate  FDG | yes | at day 23 and 40 after induction | clinical score, IHC | Citrate can reflect the inflammatory activity earlier and more sensitively than  FDG |

Table S2. Summary of imaging studies using rat models
