# Supplementary material for: EANM recommendations based on systematic analysis of small animal radionuclide imaging in inflammatory musculoskeletal diseases
Source: EJNMMI Res. 2021 Sep 6;11:85. doi: 10.1186/s13550-021-00820-8 (PMC8421483; doi:10.1186/s13550-021-00820-8)
Supplement: Supplementary file 4 — Additional file 4.Table S3. Summary of imaging studies using rabbit models. [file 13550_2021_820_MOESM4_ESM.docx]

| **Table S3.** Summary of imaging studies using rabbit models | | | | | | | | | | | |
| --- | --- | --- | --- | --- | --- | --- | --- | --- | --- | --- | --- |
| Author and DOI | Disease induction type | Strain | Imaging Study size | Study intervention | Target | Modality | Radionuclides and targeting moeities | Baseline imaging | Time after induction (observational) or intervention | Correlative outcome measure | Main imaging findings |
| Shammas et al. doi: 10.1111/hae.13090 | autologous blood induced arthropathy | New Zealand White | 2 groups, n=5-5 | observational | glucose metabolism | PET | 18F  FDG | yes | week 5 and 17 | IHC (iron-staining, synovial score and cartilage score) | FDG uptake detects reactive inflammatory process of the peri-synovial tissues associated with haemophilic arthropathy |
| Virtanen et al. doi: 10.1186/s13075-015-0826-8. | PHA induced arthritis | New Zealand White | 2 groups, n=7-7 | observational | glucose metabolism, vascular adhesion protein VAP-1 | PET | 18F,  68Ga  FDG,  Siglec-9 | no | FDG: 8-24 hrs  Siglec-9: 24 hrs | IHC | Siglec-9 detected VAP-1 positive vasculature in the mild synovitis of rabbits comparable with FDG |
| Wang et al. doi: 10.1021/jf104718g. | LPS induced acute arthritis | New Zealand White | 2 groups, N/A | interventional; Vitis thunbergii Sieb. and Zucc. var. taiwaniana Lu | glucose metabolism | PET | 18F  FDG | no | day 1 and day 7 | serum enzyme activity | PET SUV lower in treated group compared to untreated group |
| Hsieh et al. doi: 10.1016/j.jep.2010.06.042. | LPS induces acute arthritis | New Zealand White | 2 group | interventional; Ranunculaeae | glucose metabolism | PET | 18F  FDG | no | day 1, 7 | serum enzyme activity, western blot | CC6 exerted inhibitory effects on FDG PET  uptake in the joints |
| Wu et al. doi:10.1097/mnm.0b013e328329982c | *Staphylococcus aureus* induced arthritis | New Zealand big ear white | 2 groups, n=7-7 | interventional; glucose injection | DMSA uptake by glucose regulated acidosis | Scintigraphy | 99mTc  Pentavalent DMSA | no | day 14 after induction | IHC,  pH measurement | glucose loading remarkably increased the uptake  levels of 99mTc(V)-DMSA in inflammatory joint lesions. |
| Leung et al. doi:10.4077/CJP.2012.AMM113 | LPS induced arthritis | New Zealand White | 2 groups, n=3-3 | Interventional, bioceramic | glucose metabolism | PET | 18F  FDG | no | At 16 hours and 7 days after induction | Clinical score | Bioceramic reduced FDG uptake in affected joints |

Table S3. Summary of imaging studies using rabbit models
